# Comprehensive multi-omics characterization of different cuts of Dezhou donkey meat

**DOI:** 10.1016/j.fochms.2025.100267

**Published:** 2025-06-12

**Authors:** Yu Tian, Wei Zhang, Chenxi Gao, Han Wang, Junjie Wang, Shunfeng Cheng, Shuer Zhang, Min Zhang, Jianjun Li, Yujiang Sun, Wei Shen, Shuqin Liu

**Affiliations:** a College of Animal Science and Technology, Qingdao Agricultural University, Qingdao 266109, China; b Shandong Equine Genetic Resource Gene Bank, Qingdao 266109, China; c College of Life Sciences, Qingdao Agricultural University, Qingdao 266109, China; d General Station of Animal Husbandry of Shandong Province, Jinan 250100, China; e Protection of Animal Genetic Resources and Biological Breeding Engineering Research Center of Shandong Province, Jinan 250300, China; f Shandong Junchi Donkey Industry Co., Ltd., Binzhou 251903, China

**Keywords:** Donkey, Meat cuts, Nutritional components, Transcriptomics, Volatile organic compounds

## Abstract

This study conducted a detailed analysis of the nutritional composition of five different cuts of Dezhou donkey meat. Combining these findings with transcriptome data, the researchers identified region-specific gene expression patterns and uncovered key regulatory networks associated with meat quality traits across the various cuts. The genes *DLD* and *GOT1* were identified as crucial for regulating amino acid metabolism in the hind legs. In comparison, *HACD1*, *LPIN1*, and *TECR* were identified as essential regulators of fatty acid metabolism in the back. These three genes also influence the flavor of back meat by regulating tricosanoic acid, which in turn affects nonanal levels. This study presents an in-depth analysis of meat quality traits and gene expression patterns across various donkey meat cuts, highlighting the key functional genes involved. The findings offer important insights that could guide targeted breeding efforts to improve the quality of donkey meat.

## Highlights

• Comparison of basic nutritional components across five cuts of donkey meat.
• Donkey meat region-specific transcriptional atlas.
• Key genes driving amino acid and lipid metabolism in donkey meat.
• Gene-nutrient-flavor axis in donkey meat heterogeneity.
• Key flavor components across five cuts of donkey meat.

## Graphical abstract

**Figure:**
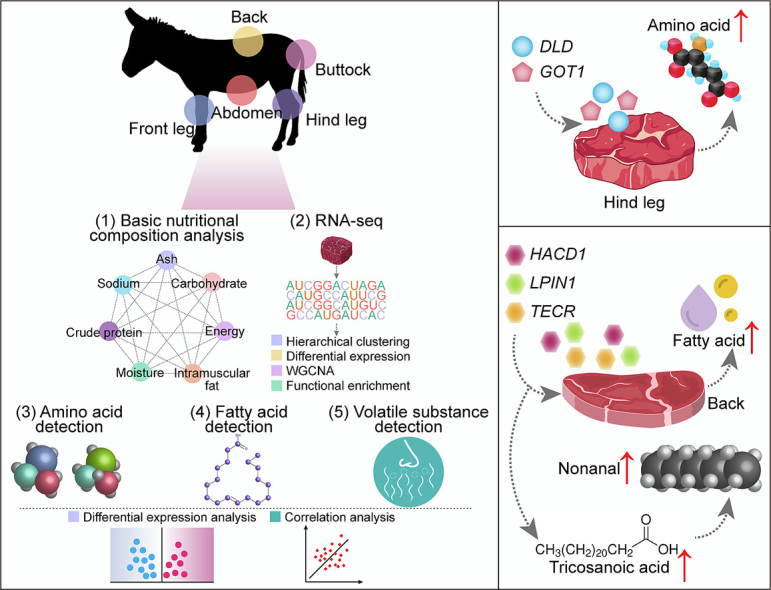
Multi-omics analysis raeveals cut-specific gene networks controlling nutritional quality and flavor in donkey meat.

## 1 Introduction

Donkey meat, which is recognized as a high-quality protein source (Polidori et al., 2021; Seyiti & Kelimu, 2021), also stands out for its high unsaturated fatty acid content, with levels that are 1.3 times, 3.1 times, 3 times and 2.4 times higher than that of beef, pork, mutton and chicken meat, respectively (Polidori et al., 2008). It is also rich in amino acids, including those essential for human health (Polidori et al., 2009). This is especially true for lysine (Lys), histidine (His), and aspartic acid (Asp), which are present at much higher levels than in other meats. Moreover, donkey meat stands out for its mineral content, with iron levels greatly surpassing those found in poultry and other common livestock (P. Polidori et al., 2009; You et al., 2008). Although the overall quality of donkey meat has been widely studied, research specifically focused on the quality traits of its different cuts remains limited.

Studying the quality of different meat cuts aligns with market demands and offers a scientific foundation for meeting the nutritional requirements of diverse consumer groups. Previous studies have shown significant variations in fatty acids, amino acids, volatile organic compounds (VOCs), trace elements, and basic nutrients among various pork cuts (Chen et al., 2021; Duan et al., 2024; Vicente & Pereira, 2024). As a result, the American Heart Association recommends pork loin for its health benefits, while pork belly has a higher risk of cardiovascular disease (Kang et al., 2024; Vicente et al., 2024). Similarly, studies have also shown considerable variations in metabolic patterns, chemical composition, fatty acid profiles, and the type and distribution of muscle fibers among different beef cuts (Oliveira et al., 2015; Yu et al., 2019), with these characteristics subsequently leading to the establishment of beef quality grades (Polkinghorne & Thompson, 2010). Therefore, studying different cuts of donkey meat is also essential for advancing and improving the donkey meat industry. Furthermore, several studies have employed high-throughput transcriptomics to explore the molecular mechanisms underlying meat quality (Fonseca et al., 2017; Meng et al., 2020; Yu et al., 2019; Yu et al., 2023). For example, Zhang et al. conducted a comparative transcriptomic analysis to reveal region-specific gene expression differences in beef, identifying key candidate genes associated with meat quality regulation (Zhang et al., 2022). Similarly, Wang et al. developed a gene-lipid-VOCs axis to analyze the regulatory network influencing pork flavor (Wang et al., 2024). However, the regulatory mechanisms underlying donkey meat quality have yet to be fully explored.

In this study, a range of meat quality assessment techniques was employed to produce detailed profiles of ash, carbohydrates, crude protein, energy, intramuscular fat, moisture, sodium, amino acids, fatty acids and VOCs across five donkey meat cuts abdomen, back, buttocks, front legs, and hind legs. These data were then integrated with transcriptomic analysis to construct a region-specific gene expression atlas and uncover the regulatory networks influencing meat quality traits in different cuts. Together, these findings offer a comprehensive understanding of donkey meat quality and highlight key functional genes that may serve as targets for improving meat characteristics.

## 2 Materials and methods

### 2.1 Ethics statement

All experimental procedures involving donkeys complied with the guidelines approved by the Animal Ethics Committee of Qingdao Agricultural University (Approval No. 2021–011).

### 2.2 Animals and sample collection

Muscle samples were collected from ten two-year-old male Dezhou donkeys in this study (Table S1). All animals were housed in clean, well-ventilated shelters and reared under consistent and controlled environmental conditions. Routine veterinary checks ensured the donkeys remained free from parasites and infectious diseases. The dietary formulation was provided by the breeding farm, and the detailed composition is presented in Table S2. The animals were obtained from the National Dezhou Donkey Breeding Farm, where they were slaughtered. Before slaughter, each donkey was individually restrained in a quiet, dimly lit setting and humanely rendered unconscious using a pneumatic stunning device. Unconsciousness was confirmed by the absence of the corneal reflex and irregular respiration. Immediately after stunning, the carotid artery and jugular vein were carefully severed with a sterile knife to ensure rapid exsanguination and permanent loss of consciousness. All procedures were performed by licensed personnel trained in humane slaughter methods, following strict animal euthanasia guidelines. Muscle samples were collected from five regions: abdomen (Musculus rectus abdominis), back (Musculus longissimus thoracis), buttocks (Musculus gluteus medius), front legs (Musculus biceps brachii), and hind legs (Musculus quadriceps femoris). The tissues were promptly frozen in liquid nitrogen for later analysis. Nutritional composition was assessed in muscle samples from all five cuts of the ten donkeys. Because of cost constraints, transcriptome sequencing was carried out on samples from the five cuts of three randomly selected donkeys. In comparison, VOCs analysis was performed on samples from the five cuts of six donkeys chosen at random. All ten donkeys belonged to the same genetic lineage to minimize variability. The three donkeys selected for transcriptome analysis were also included among the six used for VOC analysis.

### 2.3 Determination of nutritional components

Ash content was measured following the national standard GB 5009.4–2016, while crude protein was determined using the semi-micro Kjeldahl method (GB 5009.5–2010). Fat content was assessed by the Soxhlet extraction method (GB 5009.6–2016), while sodium and moisture content were analyzed according to GB 5009.268–2016 and GB 18394–2020, respectively. Based on the results, the carbohydrate and energy content were then calculated as follows: Carbohydrate = 100 – protein – intramuscular fat – moisture – ash; Energy = 17 × protein +37 × intramuscular fat +17 × carbohydrate. The nutrient content was considered to be “0” if the amount of intramuscular fat, protein and carbohydrate was ≤0.5 g/100 g.

The determination of free amino acids followed the guidelines of GB 5009.124–2016, identifying a total of 17 amino acids. Extraction was carried out following the protocol described by Lin et al. (Lin et al., 2018), with final analysis performed using an automated amino acid analyzer (Hitachi, LA8080, Japan).

Fatty acid analysis was performed according to GB 5009.168–2016, identifying a total of 37 fatty acids. The procedure involved weighing a homogenized sample, adding approximately 100 mg of pyrogallic gallic acid, a few grains of zeolite, and 2 mL of 95 % ethanol, followed by thorough mixing. Then, 10 mL of hydrochloric acid solution was added and mixed again. The mixture was hydrolyzed in a water bath at 70 °C ∼ 80 °C before analysis by gas chromatography (GC) (Agilent, 7890 A, USA) with a column length of 100 m. The temperature program was as follows: hold at 100 °C for 15 min, increase at 20 °C/min to 190 °C, and hold for 6 min, then ramp at 1 °C/min to 220 °C and hold for 7 min. The inlet temperature was set at 260 °C, with nitrogen as the carrier gas at a 1.0 mL/min flow rate. A split injection was used with a split ratio of 20:1. Detection was performed using a flame ionization detector (FID) maintained at 250 °C.

### 2.4 Transcriptomic analysis

RNA was extracted from the muscle tissue using the SPARKeasy RNA extraction kit (Sparkjade, AC0202, Shandong, China). This was followed by reverse transcription with SPARKscript II RT Plus (Sparkjade, AG0304), after which the RNA was sequenced on the MGISEQ-2000 platform. The resulting sequences were then subjected to quality control using Fastp (version 0.23.4) (Chen et al., 2018), with STAR (version 2.7.11) subsequently employed to align the reads to the donkey reference genome (Equus_asinus.ASM1607732v2.dna.toplevel.fa). Gene expression levels were quantified using featureCounts (version 2.0.6) (Liao et al., 2014). Finally, differential expression analysis was conducted with DESeq2 (version 1.40.2) (Love et al., 2014), with differentially expressed genes defined by the criteria of “Padj < 0.05; |Log_2_(FC)| ≥ 1.”

### 2.5 Weighted gene co-expression network analysis (WGCNA)

WGCNA (version 1.72–5) was carried out using the gene sets common to all five donkey meat cuts. Adjacency and topological overlap matrices (TOM) were calculated based on standard parameters. For dynamic tree cutting, a minimum module size of 100 genes was applied (Langfelder & Horvath, 2008).

### 2.6 Gene ontology (GO) enrichment analysis

GO enrichment analysis was performed on the specified gene set using g:Profiler (https://biit.cs.ut.ee/gprofiler) to identify terms that were associated with biological processes (Kolberg et al., 2023). In this case, *P*-values <0.05 were indicative of significant enrichment.

### 2.7 VOCs analysis

The meat samples were weighed and placed into headspace bottles. Then, saturated sodium chloride was added to enhance VOC extraction. The bottles were sealed tightly and incubated in a water bath at 80 °C for 20 min to reach equilibrium. A solid-phase microextraction (SPME) fiber was then inserted into the headspace, and the samples remained in the water bath at 80 °C for another 30 min. The extracted VOCs were subsequently analyzed using GC–MS (Agilent, 5975B, USA). Specifically, the extracted compounds were desorbed in the GC inlet at 250 °C using splitless mode for 5 min. Separation was performed on an HP-5MS capillary column (30 m × 0.25 mm × 0.25 μm) with helium as the carrier gas at a constant flow rate of 1.0 mL/min. The column temperature program was as follows: initial temperature at 50 °C for 2 min, ramped at 5 °C/min to 180 °C and held for 5 min, then increased at 10 °C/min to 250 °C and held for another 5 min. The mass spectrometer operated in electron ionization (EI) mode, with the ion source temperature set to 230 °C and the quadrupole temperature to 150 °C. Mass spectra were acquired in full scan mode over a mass-to-charge ratio (*m*/*z*) range of 40–600.

Differential VOCs were identified and compared based on the grouping information. The significance of each compound was first evaluated using a *t*-test to calculate the *P*-value. Thereafter, variable importance in projection (VIP) scores were calculated through multiple cross-validation. VOCs were considered significant if they met the *P*-value <0.05 and VIP > 1 criteria. The odor intensity of each VOC detected in the donkey meat samples was then assessed using the odor impact ratio (OIR), as described by Lorea et al. (Beldarrain et al., 2022). The OIR was calculated by dividing the average relative abundance of each VOC, measured across six biological replicates for each meat cut, by its corresponding odor threshold (OT). The available OT, measured in water, were then obtained from the work of Van Gemert et al. (Table S3) (Van Gemert, 2011).

### 2.8 Protein-protein interaction analysis

The STRING database (https://www.string-db.org) was used to retrieve protein-protein interaction data (Szklarczyk et al., 2023), which were subsequently used to construct cellular interaction networks with the Cytoscape software (version 3.9.1) (Shannon et al., 2003).

### 2.9 Correlation analyses

A partial least squares regression (PLSR) model was built using the pls package (version 2.8) in R to evaluate the relationship between fatty acids and VOCs. Moreover, correlation coefficients among genes, fatty acids and differentially expressed VOCs across various meat cuts were calculated using the corr.test function from the psych package in R (version 2.3.12).

### 2.10 Western blotting

Using an electric homogenizer, muscle tissue samples from various donkey cuts were homogenized in RIPA lysis buffer (Beyotime, P00113B, Shanghai, China). The extracted proteins were separated by SDS-PAGE and then transferred onto a PVDF membrane (Millipore, ISEQ00010, USA), which was blocked with 5 % BSA for 2 h at room temperature. After blocking, the membrane was incubated sequentially with primary and secondary antibodies (Table S4). Protein bands were then visualized using the Tanon 5200 imaging system (Tanon, Shanghai, China) and quantified with AlphaView SA software (ProteinSimple, California, USA).

### 2.11 RNA extraction and reverse transcriptase quantitative PCR (RT-qPCR)

Total RNA was extracted from the samples and reverse transcribed as outlined above. This was followed by RT-qPCR, performed on a CFX96 real-time PCR instrument (BioRad-CFX96, USA) using SYBR Premix Ex Taq™ fluorescent dye. The primer sequences employed for the amplification process are detailed in Table S5.

### 2.12 Statistical analysis

Data were analyzed statistically using GraphPad Prism (version 8.0.2) and presented as means ± standard error (S.E.). Differences between samples were evaluated using one-way analysis of variance (ANOVA), with significance levels defined as *P*-value <0.05 for significant and *P*-value <0.01 for highly significant differences.

## 3 Results

### 3.1 Comparison of the basic nutritional components of different donkey meat cuts

**Fig. 1:**
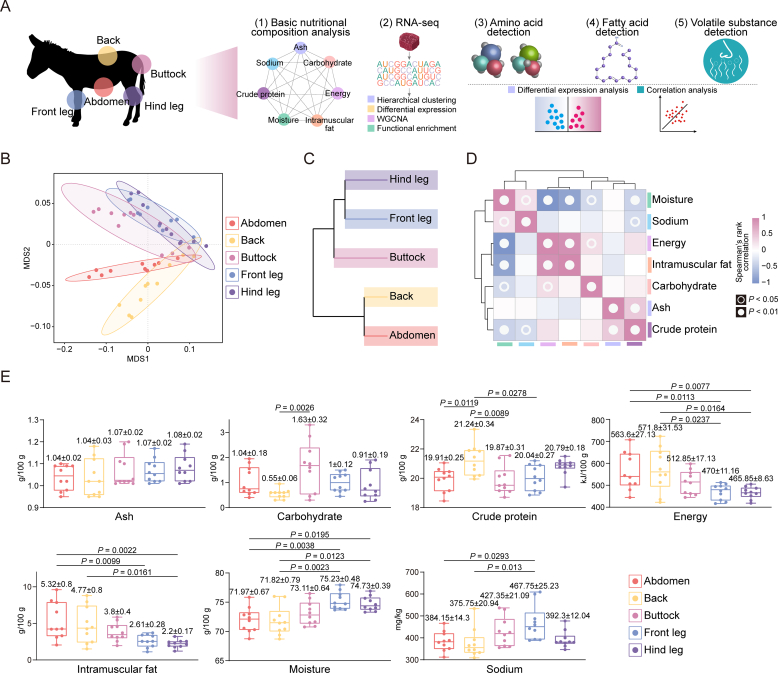
**Comparison of basic nutritional components of donkey meat from different cuts;** (A) Overall experimental design. (B) MDS plot of basic nutritional components of donkey meat from different cuts. (C) Cluster dendrogram of basic nutritional components of donkey meat from different cuts. (D) Correlation heatmap of basic nutritional components of donkey meat. (E) Descriptive statistics of ash, carbohydrate, crude protein, energy, intramuscular fat, moisture, and sodium in five donkey meat cuts. Values are shown as means ± S.E. (*n* = 10 biologically independent replicates).

Muscle tissues from the abdomen, back, buttocks, front legs, and hind legs were collected to investigate the meat quality characteristics of different donkey cuts thoroughly. A series of analyses were carried out, covering basic nutrients, amino acid composition, fat content, VOCs, and transcriptomic profiles (Fig. 1A).

This study analyzed basic nutrients, including ash, carbohydrates, crude protein, energy, intramuscular fat, moisture, and sodium. Multidimensional scaling (MDS) and hierarchical clustering revealed that the nutrient compositions of the front and hind legs were highly similar, while the back and abdomen also showed close resemblance. The nutrient profile of the buttocks was found to be more comparable to that of the legs (Fig. 1B-C). Correlation analysis indicated a positive relationship between moisture and sodium content, whereas moisture was negatively correlated with energy, intramuscular fat, carbohydrates, and crude protein. Furthermore, energy content showed significant positive correlations with intramuscular fat and carbohydrates, though no significant correlation was detected between intramuscular fat and carbohydrates. A strong positive correlation was also observed between ash and crude protein content (Fig. 1D). In the comparative analysis of nutritional components across different donkey meat cuts, no significant differences were observed in ash content among the cuts. However, carbohydrate levels in the buttocks were significantly higher than those in the back. Energy content was significantly higher in the abdomen and back compared to the front and hind legs. The abdomen also had significantly higher intramuscular fat than the front and hind legs, while the back contained more fat than the hind legs only. Moisture content was significantly higher in the front and hind legs than in the abdomen and back. Furthermore, crude protein levels were highest in the back, surpassing those in the abdomen, buttocks, and front legs. Sodium content in the front legs was significantly greater than in the abdomen and back (Fig. 1E; Table S6). These findings provide a detailed overview of the nutritional composition across different donkey meat cuts, highlighting distinct variations in their nutrient profiles.

### 3.2 Region-specific transcriptional profiles for different cuts of donkey meat

**Fig. 2:**
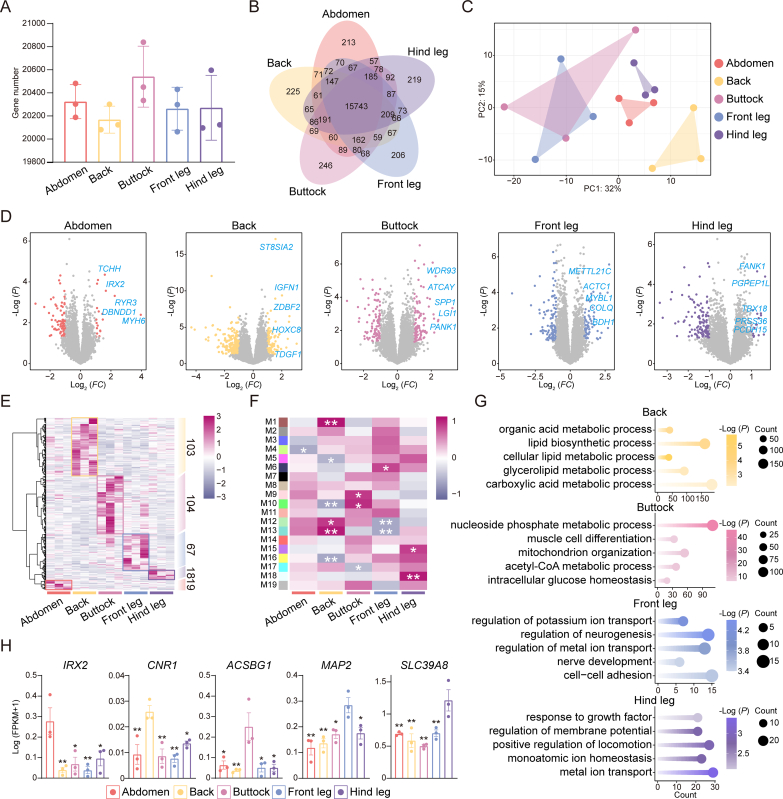
**Region-specific expression patterns analysis;** (A) The number of genes expressed in five cuts of donkey meat. (B) Number of shared and uniquely expressed genes identified in five donkey meat cuts. (C) PCA analysis of transcriptome data of donkey meat from five cuts. (D) The volcano plot showing the differentially expressed genes in donkey meat from different cuts. (E) Heatmap showing the expression patterns of specific highly expressed genes in different cuts of donkey meat. (F) Heatmap of correlations between 19 modules and 5 donkey meat cuts. * Represents significant differences (*P* < 0.05), ** Represents extremely significant differences (*P* < 0.01). (G) GO enrichment analysis results of highly correlated gene sets of donkey meat from different cuts. (H) FPKM values of genes with key functions (*n* = 3 biologically independent replicates). * Represents significant differences (*P* < 0.05), ** Represents extremely significant differences (*P* < 0.01).

Transcriptomic approaches can unveil the molecular mechanisms regulating meat quality (Fonseca et al., 2017). In this study, no significant differences were noted in the number of expressed genes across the various cuts of donkey meat, with over 20,000 genes identified in each region (Fig. 2A). Additionally, 15,743 genes were co-expressed across all five meat sections, while 213, 225, 246, 206 and 219 genes exhibited region-specific expression in the abdomen, back, buttock, front legs, and hind legs, respectively (Fig. 2B). RNA-seq datasets from all five cuts were then integrated to generate a PCA plot which revealed a distinct transcriptional profile for the back, one which was notably different from those of other cuts (Fig. 2C).

Differential expression analysis was conducted on the five RNA-seq datasets to uncover region-specific expression patterns in donkey meat. This revealed 19 highly expressed genes in the abdomen, 103 in the back, 104 in the buttocks, 67 in the front legs and 18 in the hind legs (Fig. 2D). The heatmap further demonstrated that these region-specific gene expression patterns were consistently observed across all individuals (Fig. 2E). The WGCNA was then used to identify key functional genes influencing meat quality across the different cuts. Among the 19 co-expressed gene modules identified, modules 1, 12, and 13 showed a positive correlation with the back, while modules 9 and 10 correlated positively with the buttocks. Module 6 was associated with the front legs, and modules 15 and 18 showed a positive correlation with the hind legs (Fig. 2F). GO enrichment analysis was conducted to functionally annotate the genes within these modules, with the results revealing that genes related to lipid metabolism were significantly enriched in the back, possibly due to the higher intramuscular fat content in this region. Similarly, genes associated with carbohydrate metabolism were found to be enriched in the buttocks. This genetic profile corresponds with the elevated carbohydrate content observed in this region, as quantitatively demonstrated in Fig. 1E. Collectively, these findings suggest that buttocks maintain distinctively high carbohydrate levels compared to other cuts. And, gene sets enriched in the leg region were primarily related to biological functions involving ion transport (Fig. 2G; Table S7). By integrating differential expression analysis, WGCNA, and functional annotation, we successfully identified marker genes specific to each cut of donkey meat. First, differential expression analysis was conducted by comparing the transcriptome of each individual cut to the combined transcriptomes of the remaining four cuts, using the criteria of Log_2_(FC) ≥ 1 and Padj <0.05 to identify region-specific genes. Next, WGCNA was applied to identify gene modules significantly associated with specific cuts. Genes within these modules were subjected to GO enrichment analysis, and key GO terms were selected based on statistical significance. Genes that were both significantly enriched in relevant GO terms and differentially expressed were further filtered. Subsequently, one-way ANOVA was performed to assess expression differences of these candidate genes across all cuts, and the gene with the lowest *P*-value for each cut was designated as its characteristic marker gene. The results revealed distinct expression patterns across cuts: *IRX2* exhibited significantly higher expression in the abdomen compared to the back, buttock, front leg, and hind leg. *CNR1* was predominantly expressed in the back, while *ACSBG1* showed significantly elevated expression in the buttock. *MAP2* was most highly expressed in the front leg, and *SLC39A8* showed peak expression in the hind leg (Fig. 2H). Overall, these findings demonstrated that different cuts of donkey meat exhibit distinct gene expression profiles. Based on enrichment analyses, it could subsequently be inferred that these region-specific expression patterns are closely associated with the observed variations in meat quality across different cuts.

### 3.3 Variations in the amino acid profiles of donkey meat across different cuts

**Fig. 3:**
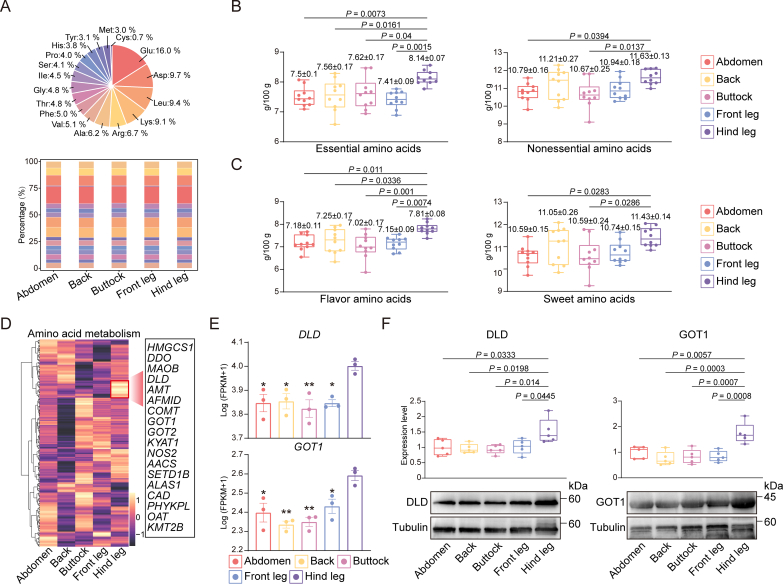
**Amino acid contents of donkey meat from different cuts;** (A) The percentage of amino acids in donkey meat (upper). The percentage of amino acids in donkey meat from different cuts (below). (B) Descriptive statistics of essential and nonessential amino acids in donkey meat from different cuts. Values are shown as means ± S.E. (n = 10 biologically independent replicates). (C) Descriptive statistics of flavor amino acids and sweet amino acids in donkey meat from different cuts. Values are shown as means ± S.E. (n = 10 biologically independent replicates). The sum of flavor amino acids was calculated as the sum of the Ala, Asp, Glu, Met, and Ser contents. The sum of sweet amino acids was calculated as the sum of the Ala, Arg, Asp, Gly, His, Lys, Pro, Ser, Thr, and Val contents. (D) Heatmap of expression patterns of genes related to amino acid metabolism in donkey meat from different cuts. (E) FPKM values of genes with key functions (*n* = 3 biologically independent replicates). * Represents significant differences (*P* < 0.05), ** Represents extremely significant differences (*P* < 0.01). (F) The protein levels of DLD and GOT1 in donkey meat from different cuts were detected. Tubulin was used as an internal control to calculate the relative protein levels (*n* = 5 biologically independent replicates).

Amino acids represent a key nutritional component of meat, and as far as the donkey meat samples were concerned, 17 amino acids were detected, with glutamate (Glu), Asp, leucine (Leu), Lys and arginine (Arg) being present in significantly higher amounts compared with other amino acids. This pattern remained consistent across various regions of donkey meat; however, the proportions of alanine (Ala), His, methionine (Met), and proline (Pro) exhibited significant variation among different cuts (Fig. 3A; Table S8). To explore the variations in amino acid profiles across the different cuts, the levels of essential amino acids, nonessential amino acids, flavor amino acids and sweet amino acids were also compared. The results showed that hind leg meat contained significantly higher amounts of essential and flavor amino acids compared with other cuts, while its non-essential and sweet amino acids content was specifically higher than in the buttocks and abdomen (Fig. 3B-C). Further comparisons of free amino acid content across the cuts revealed significant differences. In particular, as far as hind leg meat was concerned, its Ala content was significantly higher than in the abdomen, while the amounts of Arg and tyrosine (Tyr) were significantly higher than in the buttocks. Similarly, it's His content was significantly higher than in the front legs, while the Asp level was significantly higher than in the buttocks and front legs. Additionally, hind leg meat had significantly higher amounts of Glu, isoleucine (Ile), Leu, Lys, threonine (Thr) and valine (Val) compared with those of the abdomen, buttocks and front legs. Finally, the methionine (Met) content of hind leg meat was significantly higher than in the back, buttocks and front legs, while the serine (Ser) content was significantly higher than in the abdomen and buttocks (Table 1). These findings not only suggest that the hind leg meat of donkeys is particularly rich in high-quality amino acids, but they also highlight the meat's notable umami flavor, which warrants further attention.

**Table 1:** Amino acid content of donkey meat from five cuts.

Amino acid	Abdomen (g/100 g)	Back (g/100 g)	Buttock (g/100 g)	Front leg (g/100 g)	Hind leg (g/100 g)
Ala	1.12 ± 0.01^b^	1.18 ± 0.03^ab^	1.12 ± 0.03^ab^	1.15 ± 0.02^ab^	1.21 ± 0.01^a^
Arg	1.24 ± 0.02^ab^	1.27 ± 0.03^ab^	1.21 ± 0.03^b^	1.24 ± 0.01^ab^	1.33 ± 0.01^a^
Asp	1.81 ± 0.04^ab^	1.82 ± 0.04^ab^	1.76 ± 0.04^b^	1.77 ± 0.02^b^	1.92 ± 0.02^a^
Cys	0.13 ± 0.01^a^	0.14 ± 0.01^a^	0.11 ± 0.01^a^	0.13 ± 0.01^a^	0.14 ± 0.01^a^
Glu	2.94 ± 0.03^b^	3.00 ± 0.06^ab^	2.86 ± 0.07^b^	2.96 ± 0.05^b^	3.21 ± 0.04^a^
Gly	0.82 ± 0.02^a^	0.93 ± 0.05^a^	0.86 ± 0.02^a^	0.93 ± 0.07^a^	0.90 ± 0.01^a^
His	0.67 ± 0.02^ab^	0.74 ± 0.02^ab^	0.74 ± 0.03^ab^	0.65 ± 0.02^b^	0.76 ± 0.03^a^
Ile	0.82 ± 0.01^b^	0.84 ± 0.02^ab^	0.82 ± 0.01^b^	0.81 ± 0.01^b^	0.90 ± 0.01^a^
Leu	1.72 ± 0.02^b^	1.75 ± 0.03^ab^	1.71 ± 0.02^b^	1.71 ± 0.02^b^	1.84 ± 0.02^a^
Lys	1.67 ± 0.02^b^	1.72 ± 0.03^ab^	1.66 ± 0.04^b^	1.67 ± 0.02^b^	1.81 ± 0.03^a^
Met	0.56 ± 0.03^ab^	0.47 ± 0.03^b^	0.52 ± 0.03^b^	0.51 ± 0.02^b^	0.66 ± 0.02^a^
Phe	0.94 ± 0.05^a^	0.94 ± 0.06^a^	0.91 ± 0.08^a^	0.92 ± 0.04^a^	0.97 ± 0.04^a^
Pro	0.73 ± 0.02^a^	0.77 ± 0.03^a^	0.70 ± 0.02^a^	0.78 ± 0.04^a^	0.74 ± 0.01^a^
Ser	0.75 ± 0.01^b^	0.78 ± 0.02^ab^	0.75 ± 0.02^b^	0.76 ± 0.01^ab^	0.81 ± 0.01^a^
Thr	0.88 ± 0.01^b^	0.89 ± 0.02^ab^	0.87 ± 0.02^b^	0.87 ± 0.01^b^	0.95 ± 0.01^a^
Tyr	0.59 ± 0.01^ab^	0.58 ± 0.01^ab^	0.56 ± 0.02^b^	0.56 ± 0.01^ab^	0.61 ± 0.01^a^
Val	0.92 ± 0.02^b^	0.95 ± 0.02^ab^	0.92 ± 0.02^b^	0.92 ± 0.01^b^	1.00 ± 0.01^a^

The values are presented by means ± S.E. Different letters indicate significant differences among the donkey meat cuts, with *P* < 0.05.

To identify key functional genes associated with the amino acid content in donkey hind leg meat, the expression of 206 genes involved in amino acid metabolism was investigated. Hierarchical clustering pinpointed 18 genes with significantly high expression specifically in the hind leg (Fig. 3D). Furthermore, differential expression analysis revealed that *DLD* and *GOT1* were significantly upregulated in donkey hind leg meat compared to other cuts (Fig. 3E). This finding was further validated by both Western blot and RT-qPCR analyses, which consistently demonstrated significantly higher expression levels of *DLD* and *GOT1* in hind leg meat relative to other cuts (Fig. 3F; Fig. S1). Therefore, the current findings suggest that *DLD* and *GOT1* are crucial functional genes that regulate amino acid metabolism in donkey hind leg meat.

### 3.4 Identification of differentially expressed fatty acids and key regulatory genes

**Fig. 4:**
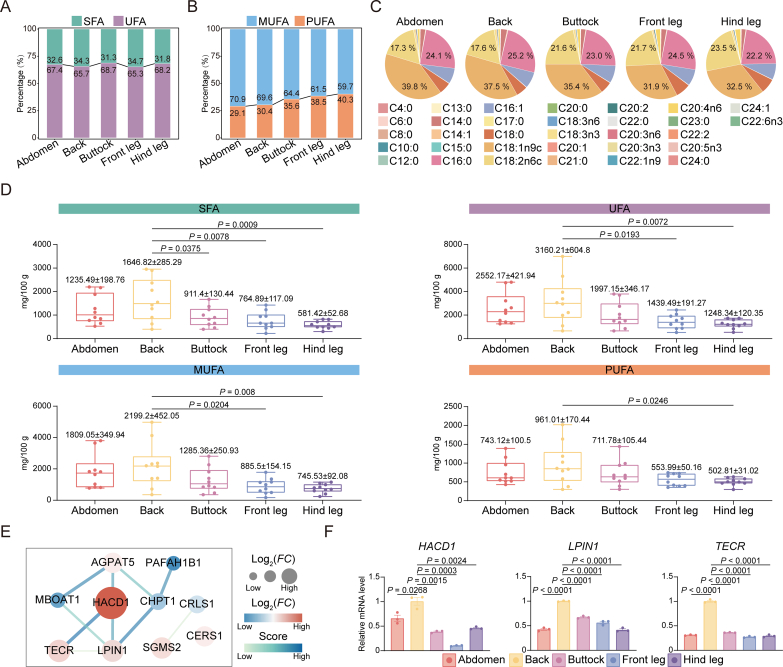
**Statistics on fatty acid composition of donkey meat;** (A) The stacking chart showing the percentage of SFA and UFA in different cuts of donkey meat. The total SFA content was calculated as the sum of the following fatty acids: C4:0, C6:0, C8:0, C10:0, C12:0, C13:0, C14:0, C15:0, C16:0, C17:0, C18:0, C20:0, C21:0, C22:0, C23:0 and C24:0. The total UFA content was calculated as the sum of the following fatty acids: C14:1, C16:1, C18:1n9c, C18:2n6c, C18:3n6, C18:3n3, C20:1, C20:2, C20:3n3, C20:3n6, C20:4n6, C20:5n3, C22:1n9, C22:2, C22:6n3, and C24:1. (B) The percentage of MUFA and PUFA in different cuts of donkey meat. The total MUFA content was calculated as the sum of the following fatty acids: C14:1, C16:1, C18:1n9c, C20:1, C22:1n9, and C24:1. The total PUFA content was calculated as the sum of the following fatty acids: C18:2n6c, C18:3n6, C18:3n3, C20:2, C20:3n3, C20:3n6, C20:4n6, C20:5n3, C22:2, andC22:6n3. (C) The percentage of fatty acids in donkey meat from different cuts. (D) Descriptive statistics of SFA, UFA, MUFA, and PUFA in donkey meat from different cuts. Values are shown as means ± S.E. (*n* = 10 biologically independent replicates). (E) Protein-protein interaction network diagram constructed based on key genes in fatty acid metabolism. (F) The expression levels of *HACD1*, *TECR* and *LPIN1* were detected by RT-qPCR, Tubulin was used as reference gene (*n* = 3 biologically independent replicates).

As consumers increasingly favor healthier dietary choices, greater focus is being placed on the fatty acid composition of meat because of its important influence on health and nutritional quality (Wood et al., 2004). This study identified 32 distinct fatty acids in donkey meat from five different cuts, including 16 saturated fatty acids (SFA) and 16 unsaturated fatty acids (UFA). The SFA content ranged from 31.3 % to 34.7 %, while the UFA content varied between 65.3 % and 68.7 % across the cuts. No significant differences were found in the SFA/UFA ratio among the cuts (Fig. 4A). The ratio of monounsaturated fatty acids (MUFA) to polyunsaturated fatty acids (PUFA) differed across the five cuts, with the abdomen and back showing a higher MUFA proportion compared to the others (Fig. 4B). Additionally, oleic acid (C18:1n9c) was the dominant fatty acid across all donkey meat cuts, comprising between 31.9 % and 39.8 % of the total fatty acid content. Palmitic acid (C16:0), accounting for 22.2 % to 25.2 % of total fatty acids, was the second most abundant fatty acid in the abdomen, back, buttocks and front legs, and ranked third in the hind legs. Linoleic acid (C18:2n6c), the most prevalent PUFA in donkey meat, was the second most abundant fatty acid in the hind legs and the third most common in the other cuts (Fig. 4C).

A comparative analysis of SFA, UFA, MUFA and PUFA levels across the five donkey meat cuts revealed significant differences. Specifically, back meat demonstrated significantly higher SFA levels than the buttocks and legs. Additionally, UFA and MUFA levels were significantly higher in back meat compared with the legs, while the PUFA content in the back also exceeded that in the hind legs (Fig. 4D). Differential analysis subsequently showed that most fatty acids were present at significantly higher levels in back meat than in other cuts, particularly when compared to the buttocks and legs (Table 2). These findings indicate that donkey back meat may provide a richer source of high-quality fatty acids. To identify the key functional genes that regulate fatty acid metabolism in donkey back meat, protein-protein interaction networks were constructed based on genes that were specifically and highly expressed in this particular cut. The analysis revealed *HACD1*, *TECR* and *LPIN1* as core functional genes (Fig. 4E), with RT-qPCR validation also confirming that these three genes exhibited high expression levels in donkey back meat (Fig. 4F). In conclusion, this study identified *HACD1*, *TECR* and *LPIN1* as crucial regulators of fatty acid metabolism in donkey back meat.

**Table 2:** Fatty acid composition in the five cuts of donkey meat.

Fatty acid	Abdomen (mg/100 g)	Back (mg/100 g)	Buttock (mg/100 g)	Front leg (mg/100 g)	Hind leg (mg/100 g)
C4:0	1.22 ± 0.22^a^	1.01 ± 0.23^a^	1.16 ± 0.24^a^	1.04 ± 0.24^a^	1.04 ± 0.25^a^
C6:0	0.04 ± 0.03^a^	ND	ND	0.08 ± 0.08^a^	ND
C8:0	0.22 ± 0.06^ab^	0.31 ± 0.07^a^	0.15 ± 0.04^ab^	0.16 ± 0.05^ab^	0.06 ± 0.03^b^
C10:0	1.66 ± 0.35^ab^	3.1 ± 0.81^a^	1.07 ± 0.21^b^	0.94 ± 0.19^b^	0.64 ± 0.11^b^
C12:0	4.78 ± 1.09^ab^	9.22 ± 2.41^a^	3.36 ± 0.66^b^	2.71 ± 0.6^b^	2.13 ± 0.37^b^
C13:0	0.23 ± 0.05^ab^	0.25 ± 0.06^a^	0.14 ± 0.03^ab^	0.12 ± 0.03^ab^	0.08 ± 0.03^b^
C14:0	88.13 ± 18.07^ab^	140.7 ± 27.84^a^	62.95 ± 11.72^b^	54.11 ± 11.99^b^	35.67 ± 4.73^b^
C14:1	8.41 ± 1.99^ab^	14.02 ± 2.9^a^	6.74 ± 1.47^b^	5.12 ± 1.16^b^	4.13 ± 0.71^b^
C15:0	4.18 ± 0.96^ab^	6.27 ± 1.48^a^	2.89 ± 0.52^ab^	2.51 ± 0.56^b^	1.83 ± 0.26^b^
C16:0	911.19 ± 152.89^ab^	1211.68 ± 215.48^a^	668.29 ± 104.36^b^	540.07 ± 91.07^b^	406.68 ± 41.29^b^
C16:1	273.7 ± 73.78^a^	362.48 ± 94.76^a^	236.46 ± 53.56^a^	167.21 ± 37.24^a^	136.96 ± 21.91^a^
C17:0	7.02 ± 1.18^ab^	9.07 ± 1.68^a^	5.16 ± 0.79^ab^	4.13 ± 0.59^b^	3.32 ± 0.31^b^
C18:0	211.61 ± 27.22^ab^	259.99 ± 41.41^a^	162.08 ± 16.88^ab^	154.41 ± 15.40^b^	126.71 ± 9.16^b^
C18:1n9c	1507.92 ± 278.42^ab^	1802.82 ± 362.73^a^	1030.97 ± 196.56^ab^	702.19 ± 115.61^b^	595.27 ± 70.43^b^
C18:2n6c	653.57 ± 88.8^ab^	847.59 ± 154.6^a^	627.8 ± 98.17^ab^	478.15 ± 45.6^ab^	430.9 ± 28.05^b^
C18:3n3	30.82 ± 6.67^ab^	48.87 ± 11.04^a^	26.76 ± 6.87^ab^	13.71 ± 2.74^b^	14.29 ± 2.55^b^
C18:3n6	0.78 ± 0.16^ab^	1.72 ± 0.58^a^	0.72 ± 0.15^ab^	0.47 ± 0.13^b^	0.58 ± 0.12^ab^
C20:0	2.65 ± 0.36^ab^	2.73 ± 0.45^a^	1.91 ± 0.33^ab^	1.97 ± 0.17^ab^	14.29 ± 4.49^b^
C20:1	13.27 ± 3.3^ab^	14.06 ± 3.1^a^	6.31 ± 1.55^ab^	5.58 ± 0.99^b^	4.13 ± 0.51^b^
C20:2	13.46 ± 3.75^a^	16.68 ± 4.15^a^	9.53 ± 1.66^a^	9.31 ± 1.85^a^	6.99 ± 1^a^
C20:3n3	1 ± 0.22^ab^	1.33 ± 0.35^a^	0.65 ± 0.16^ab^	0.39 ± 0.12^b^	3.86 ± 3.03^b^
C20:3n6	3.39 ± 0.54^a^	4.36 ± 0.82^a^	3.11 ± 0.35^a^	2.95 ± 0.36^a^	2.98 ± 0.35^a^
C20:4n6	38.56 ± 3.31^a^	38.5 ± 4.62^a^	41.7 ± 5.06^a^	47.81 ± 4.69^a^	45.04 ± 4.88^a^
C20:5n3	0.2 ± 0.09^a^	0.33 ± 0.12^a^	0.2 ± 0.11^a^	0.18 ± 0.08^a^	0.32 ± 0.1^a^
C21:0	ND	ND	ND	ND	0.01 ± 0.01
C22:0	0.76 ± 0.12^a^	0.74 ± 0.1^a^	0.59 ± 0.13^a^	0.66 ± 0.1^a^	0.49 ± 0.1^a^
C22:1n9	3.94 ± 0.44^a^	3.98 ± 0.48^a^	3.24 ± 0.4^a^	3.56 ± 0.44^a^	3.34 ± 0.41^a^
C22:2	0.14 ± 0.1^a^	0.26 ± 0.11^a^	0.1 ± 0.05^a^	0.1 ± 0.04^a^	0.08 ± 0.03^a^
C22:6n3	1.19 ± 0.15^a^	1.37 ± 0.16^a^	1.22 ± 0.11^a^	0.93 ± 0.17^a^	1.24 ± 0.11^a^
C23:0	0.47 ± 0.12^a^	0.46 ± 0.09^a^	0.44 ± 0.09^a^	0.51 ± 0.11^a^	0.41 ± 0.09^a^
C24:0	1.33 ± 0.24^a^	1.28 ± 0.24^a^	1.21 ± 0.15^a^	1.46 ± 0.51^a^	0.94 ± 0.16^a^
C24:1	1.8 ± 0.26^a^	1.83 ± 0.25^a^	1.65 ± 0.23^a^	1.85 ± 0.29^a^	1.69 ± 0.22^a^

The values are presented by means ± S.E. “ND” means not detected in the sample. Different letters indicate significant differences among the donkey meat cuts, with *P* < 0.05.

### 3.5 VOCs of five donkey meat cuts

**Fig. 5:**
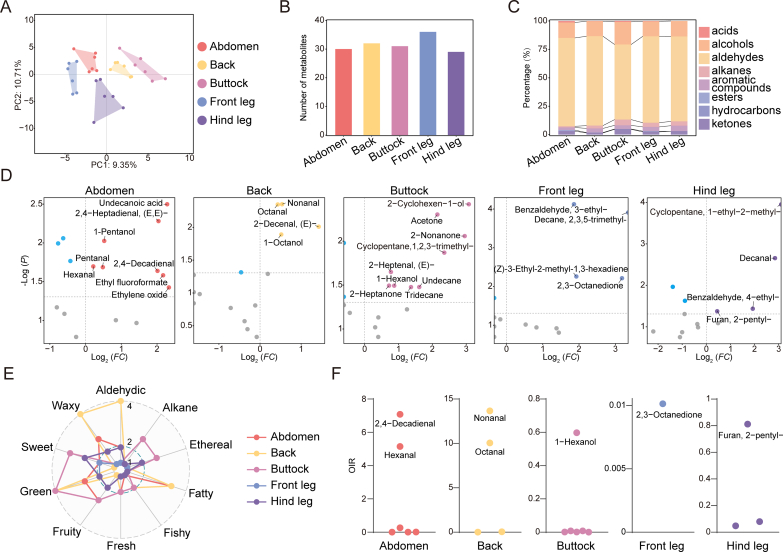
**Comparison of VOCs in different cuts of donkey meat;** (A) PLS-DA analysis of the five groups of samples. (B) Statistics on the number of VOCs detected in 5 different cuts of donkey meat. (C) The stacking chart showing the proportion of different types of VOCs in donkey meat from different cuts. (D) The volcano plot showing the different VOCs of donkey meat from different cuts. (E) Annotable VOC-enriched flavor intensity radar map for each donkey meat cut. (F) The scatter plot showing the OIR of key VOCs in donkey meat from different cuts.

Meat flavor is a critical quality and sensory attribute (Ni et al., 2022). To explore the differences in VOCs across various cuts of donkey meat, 49 VOCs, detected with GC-IMS and annotated with CAS annotations, were analyzed as potential characteristic aroma components. Partial least squares discriminant analysis (PLS-DA) subsequently confirmed distinct VOC profiles among the different cuts of donkey meat (Fig. 5A). Specifically, 30 VOCs were identified in the abdomen, 32 in the back, 31 in the buttocks, 36 in the front legs and 29 in the hind legs (Fig. 5B). Furthermore, we observed variations in the proportions of different types of VOCs across these cuts (Fig. 5C), indicating that donkey meat possesses unique flavor characteristics in different cuts.

The VOCs that were significantly different across the cuts were then identified. In the abdomen meat, the undecanoic acid, 2,4-heptadienal, (E,E)-, 1-pentanol, pentanal, hexanal, 2,4-decadienal, ethyl fluoroformate, and ethylene oxide levels were significantly elevated compared with other cuts. In contrast, back meat exhibited significantly higher levels of nonanal, octanal, 2-decenal, (E)- and 1-octanol, while buttock meat contained elevated amounts of 2-cyclohexen-1-ol, acetone, 2-nonanone, cyclopentane, 1,2,3-trimethyl-, 2-heptenal, (E)-, 1-hexanol, 2-heptanone, undecane and tridecane compared with other cuts. The front leg meat was characterized by high concentrations of benzaldehyde, 3-ethyl-, decane, 2,3,5-trimethyl-, (Z)-3-ethyl-2-methyl-1,3-hexadiene, and 2,3-octanedione. Finally, cyclopentane, 1-ethyl-2-methyl-, decanal, benzaldehyde, 4-ethyl-, and furan, 2-pentyl- were present in significantly higher concentrations in hind leg meat compared with other cuts (Fig. 5D). Based on these findings, the odor profiles of donkey meat were also characterized for different cuts. For instance, back meat was distinguished by its prominent waxy and aldehydic flavors, while buttocks meat displayed stronger alkane, ethereal and sweet notes compared with other cuts (Fig. 5E). To estimate the contribution of each VOC to the overall flavor, their OIR values were calculated. In this case, 2,4-decadienal and hexanal had higher OIR values in abdomen meat, hence indicating their significant contributions to its flavor. In contrast, for back meat, nonanal and octanal were key flavor contributors, while 1-hexanol emerged as an important flavor compound in buttock meat. Furthermore, 2,3-octanedione had the highest OIR value in front leg meat, while furan, 2-pentyl- showed higher values in hind leg meat (Fig. 5F). These results underscore the distinct flavor profiles of different donkey meat cuts and pinpoint the primary VOCs responsible for these unique aromas.

### 3.6 Construction of a regulatory network integrating genes, fatty acids, and VOCs

**Fig. 6:**
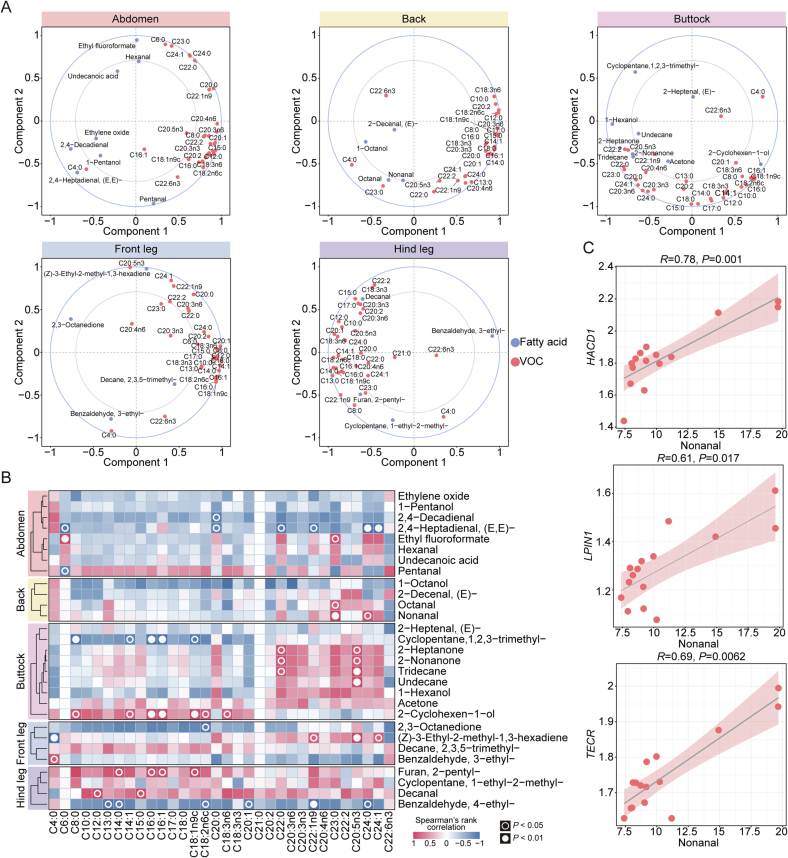
**Correlation analysis between fatty acids and key VOCs;** (A) Correlation loadings plot of fatty acids and key VOCs in the PLSR model. (B) Pearson correlation analysis heatmap of fatty acids and key VOCs. (C) Spearman correlation analysis between the expression levels of *HACD1*, *LPIN1* and *TECR* and nonanal content in donkey back meat.

Fatty acids play a crucial role in influencing the flavor of meat (Arshad et al., 2018). A correlation loading plot was constructed to reveal the fatty acids influencing the flavor of donkey meat from various cuts, associating fatty acids with specific high-abundance VOCs using the PLSR model. The inner ellipse (R^2^ = 0.5) and outer ellipse (R^2^ = 1.0) represent 50 % and 100 % of the explained variance, respectively. The results indicate a significant correlation between VOCs and fatty acids in donkey meat (Fig. 6A). Furthermore, the Pearson correlation heatmap provided detailed insights into these relationships. Specifically, in abdomen meat, 2,4-decadienal was negatively correlated with C20:0, while 2,4-heptadienal, (E,E)- showed negative correlations with C24:1, C24:0, C22:1n9, C6:0, C22:0, and C20:0. Ethyl fluoroformate was positively correlated with C23:0 and C6:0, whereas pentanal showed a negative correlation with C6:0. Regarding back meat, both octanal and nonanal showed positive correlations C23:0, with nonanal moreover correlating positively with C24:0. In buttock meat, cyclopentane,1,2,3-trimethyl- was negatively correlated with C8:0, C14:1, C16:0, C16:1, and C18:1n9c, whereas 2-heptanone, 2-nonanone and tridecane were positively correlated with C20:5n3 and C22:0. Similarly, undecane was positively correlated with C20:5n3, and 2-cyclohexen-1-ol demonstrated positive correlations with C8:0, C14:1, C16:0, C16:1, C18:1n9c, C18:2n6c and C18:3n6. In front leg meat, 2,3-octanedione had a negative correlation with C18:2n6c, while (Z)-3-ethyl-2-methyl-1,3-hexadiene was positively correlated with C24:1, C20:5n3, and C22:1n9, but negatively correlated with C4:0. Furthermore, benzaldehyde, 3-ethyl- showed a positive correlation with C4:0. For hind leg meat, furan, 2-pentyl- was positively correlated with C14:0, C16:0, C16:1 and C18:1n9c, while decanal correlated positively with C12:0 and C15:0. On the other hand, benzaldehyde, 4-ethyl- showed negative correlations with C13:0, C14:0, C18:2n6c, C20:1, C22:1n9 and C24:0 (Fig. 6B). Considering that nonanal is a key contributor to the distinctive flavor of back meat and is positively correlated with C23:0, an association analysis was conducted to explore its relationship with the main genes regulating fatty acid metabolism *HACD1*, *LPIN1*, and *TECR*. This analysis aimed to assess whether these genes are associated with the flavor profile of back meat. The results revealed significant positive correlations between the expression levels of *HACD1*, *LPIN1*, and *TECR* and the abundance of nonanal (Fig. 6C). Therefore, it is suggested that *HACD1*, *LPIN1*, and *TECR* may play crucial roles in modulating the flavor profile of back meat.

## 4 Discussion

Donkey meat is renowned for its high nutritional value and distinctive flavor, closely associated with its unique nutritional profile, particularly its rich content of amino acids and UFA (You et al., 2008). Among the different local donkey breeds in China, Dezhou donkeys are distinguished by their fast growth, excellent production performance, and consistent genetic characteristics (Wang et al., 2023). Identifying their importance, this study performed an integrative omics analysis of the abdomen, back, buttocks, front legs and hind legs of Dezhou donkeys, thoroughly characterizing the meat quality characteristics and transcriptional profiles of each cut, and identifying potential functional genes associated with meat quality.

Moisture, protein, fat, ash, and trace elements are the main nutritional components of meat. Our study found significant correlations among these components, particularly a positive association between water content and sodium levels in donkey meat. Supporting this, Débora et al. showed that adding sodium to drinking water effectively reduces drip loss in muscle tissue, underscoring the positive correlation between water and sodium content and emphasizing sodium's role in improving meat quality (de Oliveira Carvalho et al., 2023). Furthermore, comparison of donkey meat from different cuts revealed that the abdomen had a significantly higher carbohydrate content than the back. A previous research study has associated elevated carbohydrate levels with darker, firmer, and drier meat and conditions like pale, soft, and exudative meat (Ahmad et al., 2018). This indicates that abdomen meat may be more susceptible to color changes and greater drip loss than back meat. Furthermore, the intramuscular fat content of the abdomen was notably higher than that of the front and hind legs, while the fat content of the back exceeded that of the hind leg. Given that fat content positively influences the sensory quality of meat, these findings imply that abdomen and back cuts may offer superior taste compared to other cuts (Hocquette et al., 2010). Additionally, the moisture content in the front and hind legs was significantly higher than in the abdomen and back, suggesting that leg meat may have a shorter shelf life due to its higher moisture content (Ahmad et al., 2018). Significant differences were also observed in the amino acid and fatty acid profiles of donkey meat across different cuts. Our study revealed that donkey hind leg meat is particularly rich in high-quality amino acids. Notably, branched chain amino acids (BCAAs)—Leu, Ile, and Val—which are the most abundant essential amino acids, were present in significantly higher concentrations in hind leg meat compared to other cuts. Research has demonstrated that BCAAs play critical roles in glucose and lipid metabolism, protein synthesis, and the maintenance of intestinal health and immune function (Nie et al., 2018). In addition, the fatty acid content of donkey back meat was found to be significantly higher than that of other cuts. UFA is generally regarded as healthier than SFA(Bojková et al., 2020), and interestingly, myristoleic acid (C14:1) was the only UFA significantly more abundant in back meat compared to the buttocks and legs. Studies suggest that myristoleic acid may contribute to reducing obesity by activating brown adipose tissue and alleviating conditions such as non-alcoholic fatty liver disease(Li et al., 2023; Quan et al., 2020). In summary, these findings highlight the distinct nutritional characteristics of donkey meat from various anatomical regions, providing a theoretical foundation for optimizing dietary choices to address the specific nutritional preferences of different consumer groups.

It is also important to note that the flavor of donkey meat is a key quality attribute that can significantly influence consumers' long-term purchasing behavior. In this study, 2,4-decadienal and hexanal were identified as major contributors to the flavor profile of abdomen meat. Notably, Wu et al. reported that these compounds are closely associated with the rich and favorable flavor of pork from Chinese indigenous pig breeds (Wu et al., 2022). In addition, Li et al. identified hexanal as the predominant flavor compound in cooked donkey meat, suggesting that abdominal cuts may more effectively represent the characteristic flavor profile of donkey meat (Li, Sun, et al., 2024). Analysis of the back meat revealed nonanal and octanal as the predominant volatile organic compounds. These aldehydes are also abundant in Dahe black pig ham, where they contribute to its distinctive sweet, smoky, and savory flavor profile (Shi et al., 2019). The buttock meat exhibited high flavor contribution from 1-hexanol, a compound previously documented to increase substantially during the stewing process of chicken soup, thereby enhancing its aromatic complexity (Qi et al., 2017). In the front leg, 2,3-octanedione was the most significant flavor compound, known as a major aroma contributor in braised pork (Yao et al., 2024). For the hind leg, furan, 2-pentyl- emerged as a key compound, commonly associated with the characteristic flavor of cured meats (Wei et al., 2017). Together, these findings suggest that the distinct VOC profiles of different cuts of donkey meat may offer potential for diverse culinary applications and consumer segmentation based on flavor preferences.

Investigating the specific gene expression patterns in meat from different cuts provides valuable insights into the molecular mechanisms underlying meat quality characteristics. In cattle, significant differences in gene expression profiles across various cuts have been identified, and these differences are closely associated with variations in meat quality (Yu et al., 2024; Zhang et al., 2022). Similarly, our study on the transcriptional profiles of different donkey meat regions revealed comparable findings. Specifically, we identified that the gene *GOT1*, which is highly expressed in donkey hind leg meat, is closely linked to the elevated amino acid content in this region. Shen et al. demonstrated that *GOT1* is associated with serum aspartate aminotransferase levels, an enzyme that catalyzes the reversible conversion between aspartate/α-ketoglutarate and oxaloacetate/glutamate, playing a pivotal role in amino acid metabolism (Shen et al., 2011). This evidence further supports the conclusion that *GOT1* is a key functional gene regulating the amino acid content in donkey hind leg meat. Additionally, we identified candidate genes associated with fatty acid content in donkey back meat, including *HACD1*, *LPIN1*, and *TECR*. *HACD1* has been reported to be highly expressed in developing and mature striated muscle, encoding a 3-hydroxyacyl-CoA dehydratase that is involved in the biosynthesis of very long-chain fatty acids (Walmsley et al., 2017). *LPIN1* has been implicated in regulating the fatty acid profile in goat milk and is associated with the PUFA ratio in the longissimus dorsi muscle of pigs (Wu et al., 2023; Yu et al., 2013). Similarly, *TECR* is recognized as an important gene in fatty acid metabolism (Kato et al., 2024). Collectively, these findings suggest that *HACD1*, *LPIN1*, and *TECR* are candidate genes responsible for regulating the fatty acid content in donkey meat. Moreover, our study revealed a potential regulatory network “*HACD1*, *LPIN1*, *TECR*-Tricosanoic acid-Nonanal” in back meat. Among them, *HACD1* catalyzes the dehydration step in very long-chain fatty acid biosynthesis, promoting the formation of tricosanoic acid, which may subsequently undergo partial β-oxidation to generate acetyl-CoA (McDonnell et al., 2016; Sawai et al., 2017). Acetyl-CoA has been shown to play a critical role in the development of meat flavor (Li, et al., 2024). *LPIN1* regulates lipid droplet dynamics and fatty acid mobilization, indirectly influencing substrate availability for β-oxidation and the generation of aldehyde volatiles (Chen et al., 2024). *TECR* complements *HACD1* by catalyzing the terminal reduction step in the elongation pathway, collectively facilitating long-chain fatty acid synthesis (Zhou et al., 2024). Additionally, previous studies have shown that oleic acid, a long-chain fatty acid, can be oxidized to produce nonanal, suggesting that tricosanoic acid may follow a similar oxidative route (Huang et al., 2022). This proposed pathway links transcriptomic regulation with VOCs formation, offering mechanistic insights into the flavor attributes of donkey meat.

## 5 Conclusion

This study conducted a comprehensive comparative analysis of ash, carbohydrates, crude protein, energy, intramuscular fat, moisture, sodium, amino acids, fatty acids, VOCs and mRNA expression across five anatomical cuts of donkey meat. This integrative approach enabled a detailed evaluation of each cut's nutritional composition and transcriptomic profiles. A total of 311 region-specific genes, 17 amino acids, 32 fatty acids, and 49 VOCs were identified, highlighting the molecular and biochemical diversity among the cuts. Key genes involved in amino acid metabolism in the hind leg and fatty acid metabolism in the back were validated through expression analysis. Moreover, characteristic VOCs were identified for each cut, and correlation analyses associated specific fatty acids with these flavor compounds. A regulatory pathway involving “*HACD1*, *LPIN1*, *TECR*–Tricosanoic acid–Nonanal” was proposed for back meat, providing mechanistic insights into flavor development. These findings offer a comprehensive understanding of the nutritional and molecular features of donkey meat and identify critical functional genes that may serve as targets for future genetic selection aimed at improving meat quality.

## CRediT authorship contribution statement

**Yu Tian:** Writing – original draft, Software, Data curation. **Wei Zhang:** Validation, Methodology. **Chenxi Gao:** Formal analysis. **Han Wang:** Project administration. **Junjie Wang:** Conceptualization. **Shunfeng Cheng:** Resources. **Shuer Zhang:** Investigation. **Min Zhang:** Investigation. **Jianjun Li:** Resources. **Yujiang Sun:** Supervision, Funding acquisition. **Wei Shen:** Supervision, Funding acquisition. **Shuqin Liu:** Writing – review & editing, Supervision, Funding acquisition.

## Declaration of competing interest

The authors declare that they have no known competing financial interests or personal relationships that could have appeared to influence the work reported in this paper.

## Data Availability

The RNA-seq data generated in this study have been deposited in the Genome Sequence Archive (GSA, https://ngdc.cncb.ac.cn/gsa) under the accession code “CRA022933”. The results of the nutritional component analysis and volatile organic compounds profiling have been made publicly available and uploaded to GitHub. These datasets can be accessed at the following URL: https://github.com/TianYu-828/Comprehensive-multi-omics-characterization-of-different-cuts-of-Dezhou-donkey-meat.

## Appendix A Supplementary data

Supplementary material Image 1
