# Supplementary material for: Comprehensive multi-omics characterization of different cuts of Dezhou donkey meat
Source: Food Chem (Oxf). 2025 Jun 12;11:100267. doi: 10.1016/j.fochms.2025.100267 (PMC12211847; doi:10.1016/j.fochms.2025.100267)
Supplement: Supplementary file 1 — Supplementary material [file mmc1.docx]

**Supplementary Materials**

**Supplementary Table S1 Data on donkey age and height**

| Donkey ID. | Age (years) | Height (cm) |
| --- | --- | --- |
| 1  2  3  4  5  6  7  8  9  10 | 2  2  2  2  2  2  2  2  2  2 | 133.5  131  131  133  127  135  139  129  128.5  126.5 |

**Supplementary Table S2 Dietary formulations profile**

| Item | Content (%) |
| --- | --- |
| Corn  Soybean  Bran  Corn straw  Wheat straw  Vitamin  Mineral  Salt | 14.50  3.80  0.50  40.00  40.00  0.50  0.50  0.20 |

**Supplementary Table S3 Odour threshold of major volatile organic compounds**

| Volatile organic compounds | CAS | Odour threshold |
| --- | --- | --- |
| Undecanoic acid  2,4-Heptadienal, (E,E)-  1-Pentanol  Pentanal  2,4-Decadienal  Hexanal  Nonanal  Octanal  2-Decenal, (E)-  1-Octanol  Acetone  2-Nonanone  2-Heptenal, (E)-  1-Hexanol  Undecane  2-Heptanone  2,3-Octanedione  Decanal  Benzaldehyde, 4-ethyl-  Furan, 2-pentyl- | 000112-37-8  004313-03-5  000071-41-0  000110-62-3  025152-84-5  000066-25-1  000124-19-6  000124-13-0  003913-81-3  000111-87-5  000067-64-1  000821-55-6  018829-55-5  000111-27-3  001120-21-4  000110-43-0  000585-25-1  000112-31-2  004748-78-1  003777-69-3 | 10 mg/kg  0.0154 mg/kg  0.1502 mg/kg  0.012 mg/kg  0.0003 mg/kg  0.005 mg/kg  0.0011 mg/kg  0.0008 mg/kg  1.8 mg/kg  0.05 mg/kg  100 mg/kg  0.05 mg/kg  0.051 mg/kg  0.007 mg/kg  0.62 mg/kg  0.68 mg/kg  0.05 mg/kg  0.005 mg/kg  0.013 mg/kg  0.0048 mg/kg |

**Supplementary Table S4** **Antibodies used in this paper**

| Primary antibodies | Vendor | Dilution | Source |
| --- | --- | --- | --- |
| β-Tubulin  GOT1  DLD | ABclonal (AC008)  ABclonal (A11363)  ABclonal (A5220) | 1:1000  1:2000  1:1000 | Rabbit  Rabbit  Rabbit |

| Secondary antibodies | Vendor | Dilution | Source |
| --- | --- | --- | --- |
| HRP-conjugated goat anti-rabbit IgG | ABclonal (AS014) | 1:5000 | Goat |

**Supplementary Table S5 Primers used for RT-qPCR**

| Gene | Forward primer sequence | Reverse primer sequence |
| --- | --- | --- |
| *β-Tubulin*  *HACD1*  *TECR*  *LPIN1*  *GOT1*  *DLD* | CAAGATCAAGACCGGCGTGG  CCTACTTCTGTGCTCGTGGC  GCGGGAGAAGCTGTGTTTCC  CTTCCGTGCTTCAGACAGTCCTTC  CTCTCTCGCCATGACGTCTC  TATTCTGGGACCAGGTGCTG | TGCAGGATCTCTGCCTGCTT  CACTCTCCTCATTCTGGATTGGT  TACCACTGCGGATGGCTCTT  TGGCAGCTTGTGGCAATTCTCC  TCATCCGTGCGATAAGCTCC  GATGCGCGTGACAGACTCTA |

**Supplementary Table S6 Comparison of the basic nutritional components of donkey meat from five cuts**

|  | Abdomen | Back | Buttock | Front leg | Hind leg |
| --- | --- | --- | --- | --- | --- |
| Ash (g/100 g)  Carbohydrate (g/100 g)  Crude protein (g/100 g)  Energy (kJ/100 g)  Intramuscular fat (g/100 g)  Moisture (g/100 g)  Sodium (mg/kg) | 1.04±0.02^a^  1.04±0.18^ab^  19.91±0.25^b^  563.6±27.13^a^  5.32±0.8^a^  71.97±0.67^b^  384.15±14.3^b^ | 1.04±0.03^a^  0.55±0.06^b^  21.24±0.34^a^  571.8±31.53^a^  4.77±0.8^ab^  71.82±0.79^b^  375.75±20.94^b^ | 1.07±0.02^a^  1.63±0.32^a^  19.87±0.31^b^  512.85±17.13^ab^  3.8±0.4^ab^  73.11±0.64^ab^  427.35±21.09^ab^ | 1.07±0.02^a^  1.00±0.12^ab^  20.04±0.27^b^  470±11.16^b^  2.61±0.28^b^  75.23±0.48^a^  467.75±25.23^a^ | 1.08±0.02^a^  0.91±0.19^ab^  20.79±0.18^ab^  465.85±8.63^b^  2.2±0.17^b^  74.73±0.39^a^  392.3±12.04^ab^ |

The values are presented by means ± S.E. Different letters indicate significant differences among the donkey meat cuts, with *P* < 0.05.

**Supplementary Table S7 GO enrichment analysis results of highly correlated module gene sets in different cuts**

| Cuts | GO term | -Log(*P*) | Count |
| --- | --- | --- | --- |
| Back | organic acid metabolic process  lipid biosynthetic process  cellular lipid metabolic process  glycerolipid metabolic process  carboxylic acid metabolic process | 3.7  3.7  5.8  2.6  2.3 | 41  162  38  91  186 |
| Buttock | nucleoside phosphate metabolic process  muscle cell differentiation  mitochondrion organization  acetyl-CoA metabolic process  intracellular glucose homeostasis | 46  6.8  9  8.7  7 | 110  33  54  37  19 |
| Front leg | regulation of potassium ion transport  regulation of neurogenesis  regulation of metal ion transport  nerve development  cell−cell adhesion | 4.2  4.4  3.8  3.4  3.5 | 7  14  13  6  15 |
| Hind leg | response to growth factor  regulation of membrane potential  positive regulation of locomotion  monoatomic ion homeostasis  metal ion transport | 2.1  2.8  2.9  2.8  3.4 | 21  21  27  23  29 |

**Supplementary Table S8 Amino acid ratios of donkey meat from five cuts**

| Amino acid | Abdomen (%) | Back (%) | Buttock (%) | Front leg (%) | Hind leg (%) |
| --- | --- | --- | --- | --- | --- |
| Ala  Arg  Asp  Cys  Glu  Gly  His  Ile  Leu  Lys  Met  Phe  Pro  Ser  Thr  Tyr  Val | 6.12±0.03^b^  6.75±0.05^a^  9.88±0.16^a^  0.70±0.05^a^  16.07±0.11^a^  4.50±0.07^a^  3.67±0.09^ab^  4.50±0.05^a^  9.42±0.06^a^  9.12±0.07^a^  3.07±0.17^a^  5.13±0.08^a^  3.98±0.06^ab^  4.09±0.02^a^  4.79±0.02^a^  3.22±0.05^a^  5.02±0.08^a^ | 6.31±0.04^a^  6.78±0.06^a^  9.69±0.04^a^  0.72±0.06^a^  16.01±0.09^a^  4.96±0.21^a^  3.94±0.09^ab^  4.48±0.04^a^  9.34±0.07^a^  9.17±0.09^a^  2.48±0.12^b^  4.99±0.09^a^  4.07±0.09^ab^  4.15±0.03^a^  4.75±0.03^a^  3.11±0.04^a^  5.05±0.04^a^ | 6.21±0.03^ab^  6.68±0.04^a^  9.75±0.04^a^  0.62±0.03^a^  15.82±0.18^a^  4.74±0.10^a^  4.07±0.18^a^  4.52±0.05^a^  9.46±0.07^a^  9.18±0.08^a^  2.89±0.18^ab^  5.03±0.05^a^  3.88±0.05^ab^  4.16±0.05^a^  4.81±0.02^a^  3.08±0.04^a^  5.11±0.07^a^ | 6.28±0.07^ab^  6.78±0.04^a^  9.65±0.09^a^  0.71±0.05^a^  16.13±0.19^a^  5.07±0.34^a^  3.53±0.11^b^  4.41±0.07^a^  9.34±0.11^a^  9.09±0.09^a^  2.79±0.12^ab^  5.00±0.08^a^  4.24±0.22^a^  4.12±0.02^a^  4.73±0.06^a^  3.08±0.05^a^  5.05±0.08^a^ | 6.13±0.02^b^  6.72±0.03^a^  9.70±0.03^a^  0.69±0.04^a^  16.24±0.12^a^  4.54±0.08^a^  3.86±0.15^ab^  4.56±0.04^a^  9.32±0.04^a^  9.16±0.07^a^  3.36±0.12^a^  4.92±0.04^a^  3.76±0.06^b^  4.10±0.03^a^  4.79±0.02^a^  3.11±0.04^a^  5.07±0.04^a^ |

The values are presented by means ± S.E. Different letters indicate significant differences among the donkey meat cuts, with *P* < 0.05.


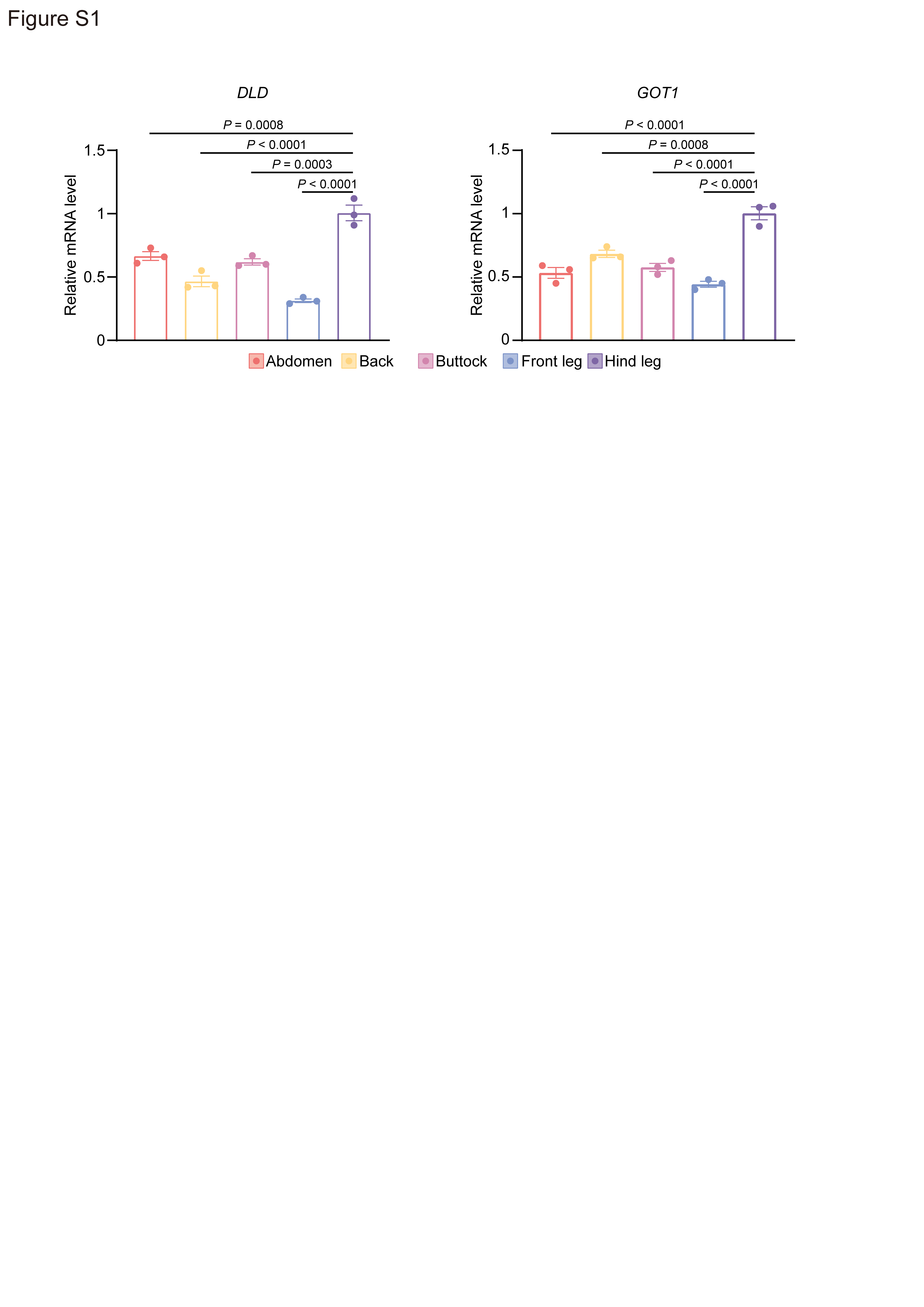


**Supplementary Fig. S1. Analysis of *DLD* and *GOT1* expression levels in donkey meat from different cuts**

The expression levels of *DLD* and *GOT1* were detected by RT-qPCR, Tubulin was used as reference gene (n = 3 biologically independent replicates).
